# Outcome of SARS-CoV-2 infection among patients with common variable immunodeficiency and a matched control group: A Danish nationwide cohort study

**DOI:** 10.3389/fimmu.2022.994253

**Published:** 2022-09-23

**Authors:** Terese L. Katzenstein, Line D. Rasmussen, Camilla Helberg Drabe, Carsten Schade Larsen, Ann-Brit Eg Hansen, Mette Stærkind, Lene Surland Knudsen, Christian Holm Hansen, Niels Obel

**Affiliations:** ^1^Department of Infectious Diseases, Copenhagen University Hospital Rigshospitalet, Copenhagen, Denmark; ^2^Department of Infectious Diseases, Odense University Hospital, Odense, Denmark; ^3^Department of Infectious Diseases, Aarhus University Hospital, Aarhus, Denmark; ^4^Department of Infectious Diseases, Copenhagen University Hospital Hvidovre, Copenhagen, Denmark; ^5^Faculty of Health Sciences, University of Copenhagen, Copenhagen, Denmark; ^6^Department of Infectious Diseases, Aalborg University Hospital, Aalborg, Denmark; ^7^Department of Medicine, Zealand University Hospital, Roskilde, Denmark; ^8^Department of Infectious Disease Epidemiology and Prevention, Statens Serum Institut, Copenhagen, Denmark

**Keywords:** severe adult respiratory coronavirus-2 (SARS-CoV-2), severe novel coronavirus 2019 (COVID-19), inborn errors of immunity (IEI), common variable immunodeficiency (CVID), clinical outcome

## Abstract

The risk of severe adult respiratory coronavirus-2 (SARS-CoV-2) infection and the course of the infection among individuals with common variable immunodeficiency (CVID) relative to the general population have been a matter of debate. We conducted a Danish nationwide study comparing the timing of SARS-CoV-2 vaccination, the risk of first confirmed SARS-CoV-2 infection, re-infection, and the outcome of infection among individuals with CVID relative to an age- and gender matched control group. Cox regression was used to calculate incidence rate ratios. The CVID patients received SARS-CoV-2 vaccinations earlier than those included in the population control group. Even so, the risks of both first infection and re-infection were increased among the individuals with CVID. The CVID group also had increased risk for hospital contacts due to SARS-CoV-2 infection relative to the general population. However, reassuringly, the risk of mechanical ventilation and death did not differ between the groups, but the numbers were low in both groups, making the estimates uncertain. Though this is the largest study to investigate the risk of SARS-CoV-2 infections and outcomes hereof among individuals with CVID relative to the general population, we cannot rule out minor differences in severity, which might only be detectable with an even larger sample size.

## Introduction

The course of severe acute respiratory syndrome coronavirus 2 (SARS-CoV-2) infection among individuals with inborn errors of immunity (IEI) relative to that seen in the general population has been a matter of debate. Individuals with IEI might be more prone to infection and at increased risk of fulminant infections. On the other hand, it has been claimed that the contribution of inflammation in severe novel coronavirus 2019 (COVID-19), the so-called cytokine storm, might be attenuated in individuals with some types of IEI (1). Counterintuitively, IEI might thereby protect against severe COVID-19 (1, 2). Case series and case reports reporting both mild (3–5) and more severe courses of COVID-19 among individuals with IEI (6–9) have been published. In a recent review of SARS-CoV-2 infection among individuals with IEI, Tangye et al. (10) found that the severity of disease and case fatality rate were not considerably different from that observed for the general population. In contrast, genetic variants (as well as auto-antibodies) affecting parts of the innate immune system have been associated with increased risk of fulminant SARS-CoV-2 infection (10–12). Given the enormous heterogeneity among patients with IEI, the variation in target populations in the studies (children *vs*. adults *vs*. both groups), and the substantial differences in constellation of IEIs in different populations (3, 6, 7, 9, 13–17), it is perhaps not surprising that the messages have been mixed.

Even among more narrowly defined IEI groups, the reported outcomes have varied. Early case reports indicated that patients with agammaglobulinemia might fare better after SARS-CoV-2 infection than individuals with common variable immunodeficiency (CVID) (18), a notion that has subsequently been challenged (19).

We have previously reported relative benign courses of SARS-CoV-2 infections among 11 Danish CVID patients (20). This concurs with reports from New York (21), Italy (22), and Israel (5), whereas other studies have found high mortality rates among IEI patients including CVID patients (8).

We used Danish national health registers to perform a nationwide, population-based study of the impact of SARS-CoV-2 infection in individuals with CVID compared to an age- and gender-matched cohort from the general population. The objectives were to compare the timing of vaccinations, and the risk of infection, hospital contact, hospitalization, and death among individuals with CVID relative to those of the general population.

The availability of a unique personal identification number assigned to all Danish residents and accessibility to national database with relevant information makes Denmark an ideal setting for performing studies of this type.

## Materials and methods

We performed a nationwide, population-based, matched cohort study comparing risk of SARS-CoV-2 infection and outcomes hereof among individuals with CVID relative to the general population. In Denmark, all treatments for COVID-19 are hospital-based. The only exception from this is Molnupiravir^®^, which could be prescribed by general practitioners. However, the usage of this treatment option has been very limited.

### Settings

In Denmark, monitoring of adult individuals with IEI are centralized to one to two Departments of Infectious Diseases per region (there are five regions in Denmark). The first case of SARS-CoV-2 infection in Denmark was detected on 27 February 2020. Vaccination was initiated ultimo December 2020. Individuals assumed to be at increased risk of severe SARS-CoV-2 infection, including individuals with IEI, were among the first to be offered SARS-CoV-2 vaccination (23). Comirnaty^®^ from Pfizer/BioNTech and Spikevax^®^ from Moderna were the first vaccines to be approved. As of 1 May 2022, 78% of the 5.8 million living in Denmark have been anti-SARS-Cov-2 vaccinated at least once. In Denmark, the vaccination program has been primarily with the mRNA-based vaccines from Pfizer (Comirnaty^®^) and Moderna (Spikevax^®^) with substantially fewer receiving the vector-based vaccines from AstraZeneca (Vaxzeria^®^) or Janssen (COVID-19 Vaccine Janssen). In Denmark, both vaccination and testing for as well as treatment of SARS-CoV-2 infection are provided free-of-charge.

### Data sources

We used the unique personal identification number (PIN) assigned to all Danish residents at birth or upon immigration to combine register data as described previously (24).

We used data from the Danish Civil Registration System (CRS) that has recorded information on all Danish residents since 1968. Using the PIN, we extracted information on vital status, date of birth, sex, date of immigration and emigration, and date of death (25). The register also includes date of death if an individual dies outside of hospital. Information regarding SARS-CoV-2 vaccination types and timing was retrieved from the Danish Vaccination Register containing individual-level information on all vaccines given in Denmark (26).

From the national COVID-19 surveillance system at Statens Serum Institut, data on results of SARS-CoV-2 PCR and antigen testing were obtained. The registry includes results of all SARS-CoV-2 tests performed at both public and private testing facilities (27).

Data on morbidity were extracted from the Danish National Patient Register (DNPR). The DNPR includes information on date of admission, date of discharge, diagnoses, and procedures recorded using the International Classification of Diseases classification system (ICD-10) and the Danish Health Care Authorities’ Classification System (SKS) (28). Charlson comorbidity score was calculated based on ICD-10 coding (29).

### Study population

We included all individuals ≥18 years who were alive and living in Denmark on the date of study inclusion and were registered with one of the following ICD-10 codes: D830, D838, or D839 three times or more. For each patient, we randomly extracted eight population controls from the general population, matched on gender and date of birth (born within 7 days from the CVID patient). All population controls had to be alive and residing in Denmark on the date of study inclusion. Date of study inclusion was 1 March 2020 or the date the individual was registered with three CVID diagnosis, whichever came last. Population controls had the same date of study inclusion as the patient, to whom they were matched.

### Outcomes

Outcomes were calculated as time to the following events:

*Date of first positive SARS-CoV-2 test:* First date the individual was registered in the national COVID-19 surveillance system with a positive PCR or antigen test for SARS-CoV-2.

*Date of second positive SARS-CoV-2 test*: Date of positive SARS-CoV-2 testing by either PCR or antigen minimum 90 days after first positive SARS-CoV-2 testing.

*Date of immunization coverage by two or three SARS-CoV-2 vaccinations*: Date of the individual’s second/third vaccination as registered in the Danish Vaccination Register. An individual was considered to have the full effect 14 days after the date of vaccination.

*Date of first hospital contact with a diagnosis of COVID-19*: First date of hospital contact (inpatient or outpatient) with a diagnosis in the DNPR of either ICD10 codes B342 (corona virus infection, unspecified sites) and/or B972 (severe corona virus infection) and/or procedures involving mechanical ventilation using the SKS code BGDA (ventilator treatment and other assisted ventilation/breathing), combined with a positive test for SARS-CoV-2 within 14 days of diagnosis.

*Date of first hospitalization with severe COVID-19*: First date an individual was hospitalized (irrespective of length of hospitalization) with a diagnosis in the DNPR of either ICD10 codes B972 and/or procedures involving mechanical ventilation (SKS code: BGDA) combined with a positive SARS-CoV-2 test within 14 days of diagnosis.

*First date of hospitalization after first positive SARS-CoV-2 test:* First date of hospitalization for more than 24 h after first positive SARS-CoV-2 test (irrespective of clinical diagnosis).

Statistical analyses

We calculated incidence rate ratios (IRRs) in individuals with CVID *vs*. individuals in the matched population control cohort for the outcomes described above. Time was calculated from date of study inclusion until date of emigration, death, 1 May 2022, or the outcome of interest. In analyses of time to vaccination, time was calculated from 1 January 2021. In analyses of time to hospitalization and death after a first positive SARS-CoV-2 test, time was calculated from date of first positive test.

We computed Kaplan–Meier life tables using calendar-time as time scale. In analyses of time to hospital contact after first positive SARS-CoV-2 test, we used days after a positive test as time scale. We used Cox regression to calculate IRR as estimates of relative risks. In analyses of time to hospital contact, after a SARS-CoV-2 test and vaccine effects, estimates were adjusted for age and gender.

Data were analyzed using STATA 14 statistical software (30).

### Ethical considerations

This study was performed as a national surveillance study under the authority task of the Danish national infectious disease control institute, Statens Serum Institut. The study was approved by the Danish Data Protection Agency (permission no 21/04383). According to Danish regulations, national surveillance activities as well as studies solely relying on register information do not require individual consent or approval from an ethics committee.

## Results

### Characteristics of the study population

The study population consisted of 313 individuals with CVID and 2,504 controls from the general population. The median age of the CVID group was 48 (33–64) years. Fifty-two percent were female, with the vast majority born in Denmark. The Charlson comorbidity score differed between the two groups (**Table 1**).

**Table 1:** Study population characteristics, CVIOD and population controls in Denmark, March 1, 2020 – May 1, 2022.

	CVID	Controls
Number	313	2,504
Age, median (IQR)	48.3 (32.9-64.4)	48.3 (32.9-64.4)
Female gender, N (%)	164 (52.4)	1,312 (52.4)
Country of origin,Denmark, N (%)	299 (99.5)	2,280 (91.1)
Charlson’s comorbidity score index:		
Low (score = 0), N (%)	96 (30.7)	1,983 (79.2)
Medium (score= 1-2), N (%)	149 (47.6)	411 (16.4)
High (score > 2), N (%)	68 (21.7)	110 (4.4)
Total person-years of follow-up (PYR )	494.0	4,066.7

N, number; CVID, Common variable immunodeficiency; IQR, interquartile range.

The study included 494 person-years of follow up in the CVID group and 4,067 in the corresponding control group (**Table 1**).

### Time to vaccination

**Figure 1:**
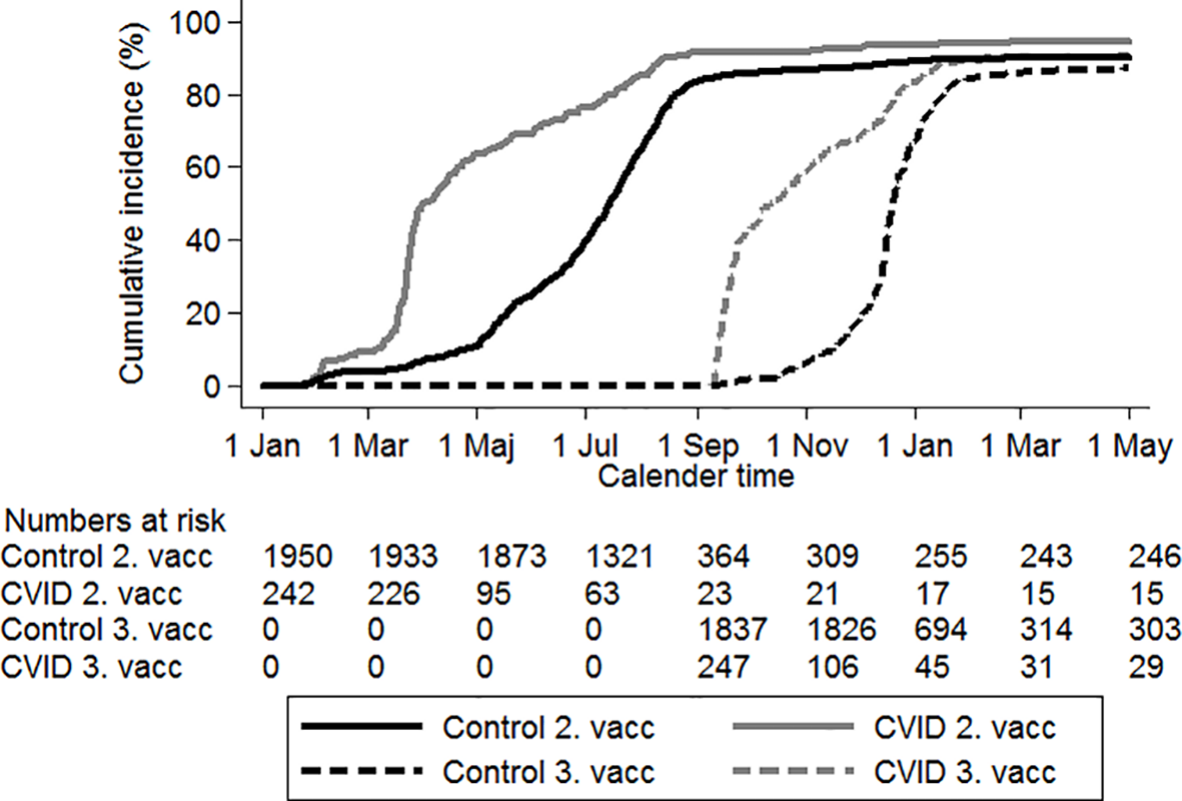
Time to second and third SARS-COV-2 vaccination. Black lines: population controls, gray lines: CVID patients. Solid lines: second vaccination, dotted lines: third SARS-CoV-2 vaccination. Time starts on January 1, 2020 and ends on May 1, 2022.

In accordance with National Danish guidelines (23), the CVID group had received two and three vaccinations prior to the age-matched controls (**Figure 1**).

### Time to confirmed SARS-CoV-2 infection (PCR or antigen test)

**Figure 2:**
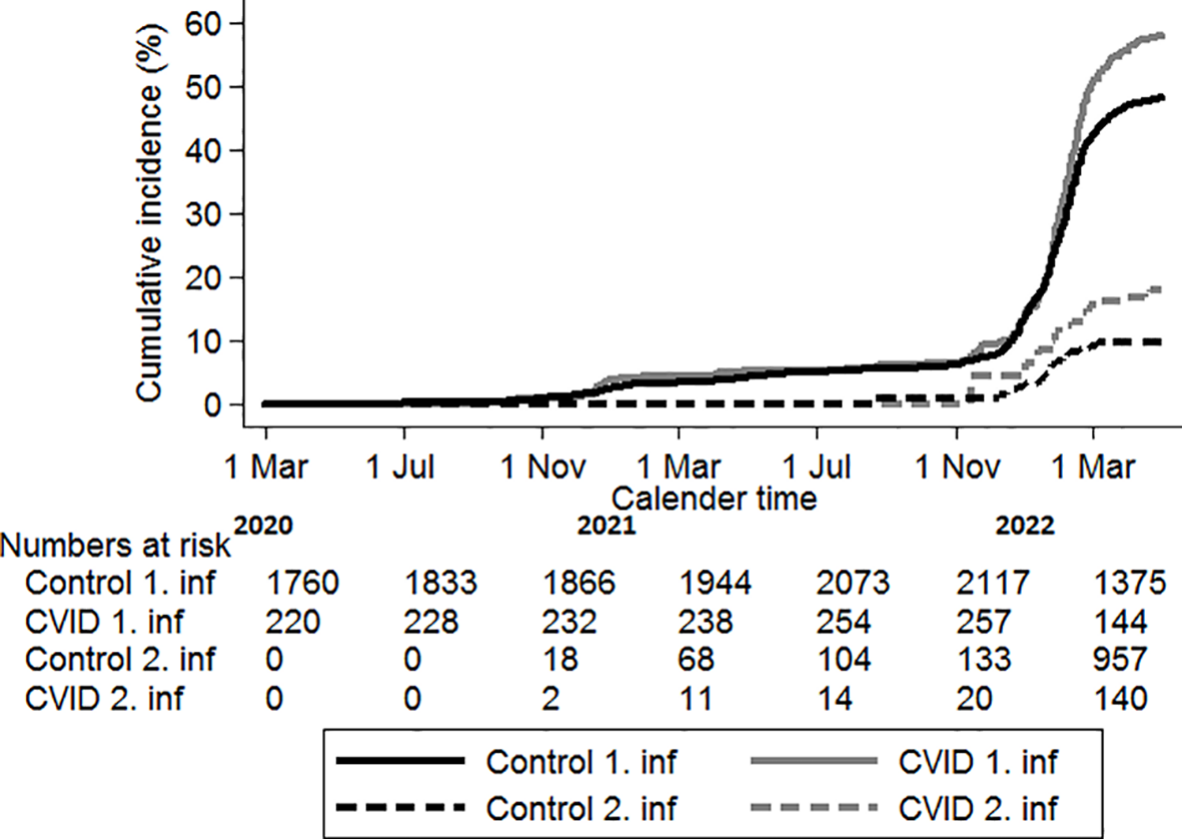
Time to first and second positive SARS-CoV-2 test. Solid lines: first positve test, dotted line: second positive SARS-CoV-2 test. Black lines: population controls, gray lines: CVID patients.

The time to first positive SARS-CoV-2 test differed slightly between the CVID and the control group [IRR 1.3 (95% CI, 1.1–1.5)] (**Table 2**). However, as illustrated in **Figure 2**, the difference in risk seemed to be mainly carried by a higher risk of testing SARS-CoV-2 positive after 1 January 2022. Before 1 January 2022: IRR 1.1 (95% CI, 0.8–1.5) *vs*. after 1 January 2022: IRR 1.4 (95% CI, 1.1–1.6).

**Table 2:** Risk of first positive SARS-CoV-2 test, vaccination, hospitalization and death in CVID patients vs. population controls in Denmark, March 1, 2020 – May 1, 2022.

	CVID patients	Observation time	Controls	Observation time	Unadjusted IRR 195% en	Adjusted IRR* 195% en
All individuals	N	PYR	N	PYR		
First occurrence of a positive test for COVID-19^1^	164	494	1,114	4,067	1.3 (1.1-1.5)	1.6 (1.3-1.9)
Second occurrence of a positive test for COVID-19^1^	15	56	51	390	2.1(1.2-3.8)	2.1(1.1-4.1)
Hospital contact^1^	45	539	16	4,463	24.5 (13.9- 43.4)	20.3 (10.6- 38.7)
Hospitalization with severe COVID-19^1^	4	551	2	4,469	16.2 (3.0-88.5)	10.7 (1.5-78.5)
SARS-CoV-2 positive patients only		PYR		PYR	IRR	aIRR**
Time to hospitalization > 24 hours after a positive test^2^	11	32	17	239	4.8 (2.2-10.2)	2.6 (1.1-6.1)
Death following a positive SARS-CoV-2 test^1^	1	242	4	35	1.7 (0.2-15.2)	0.4 (0.0-3.8)

CVID, Common variable immunodeficiency; PY, Person years of follow-up; incidence Rate Ratio, Adjusted NR: 9%: Confidence interval.

^1^Calendar time as time axis time since SARS-CoV 2 positive as time axis.

*Adjusted for charlsons Comorbidity index, categorised.

**Adjusted for age, gender and Charlson's Comorbidity Index.

Similarly, for timing to re-infection: IRR 2.1 (95% CI, 1.2–3.8) (**Figure 2**, **Table 2**).

### Testing frequencies among CVID and controls during the study period

The frequencies of SARS-CoV-2 testing throughout the study period differed between the two populations. During the initial study period, the individuals with CVID had increased testing frequencies, while during the period from March to August 2021, the population controls had higher testing rates. During the latter part of the study period, the testing frequencies only differed slightly (**Appendix Figure 1A**).

### Time to hospital contact/hospitalization due to COVID-19

**Figure 3:**
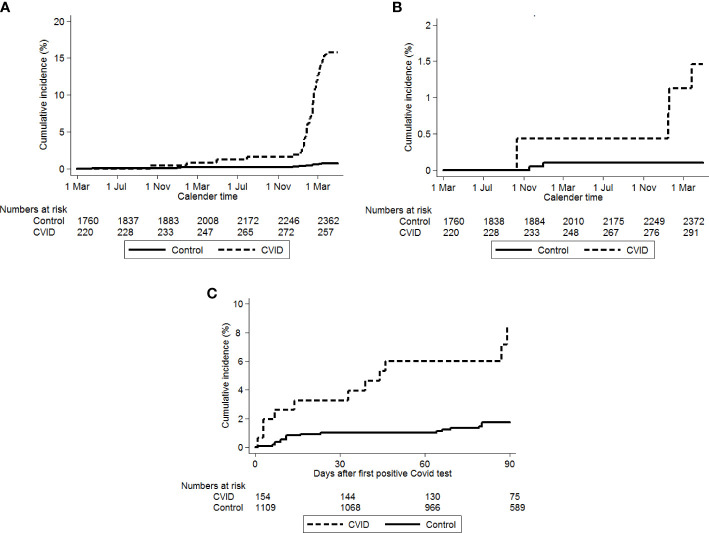
**(A)** First hospital contact with COVID-19. Solid line: population controls, dotted line: CVID patients. Time starts on 1 March 2020. **(B)** Time to first hospitalizations with severe COVID-19, Solid line: population cointrol, dotted line: CVID patients. Time starts on 1 March 2020. **(C)** Time to hospitalization after the first positive SARS-CoV-2 test. Solid line: population controls, dotted line: CVID patients. Time starts on date of first positive SARS-CoV-2 test.

A total of 45 individuals with CVID had hospital contact with COVID-19, although only 4 were hospitalized with severe COVID-19 (**Table 2**). The risk of both were increased [IRR 24.5 (95% CI, 13.9–43.4) and IRR 16.2 (95% CI, 3.0–88.5), respectively]. Individuals with CVID had an increased risk of hospitalization (>24 h) after testing SARS-CoV-2 positive relative to the control group, IRR 4.8 (95% CI, 2.2–10.2) (**Figure 3**, **Table 2**). As illustrated in **Figure 3**, the large difference in risk of first hospital contact seemed to be mainly carried by a higher risk after 1 January 2022.

Very few individuals in both groups (the CVID and population control group) were hospitalized with severe COVID-19 with need of mechanical ventilation (data not shown).

### Time to death after testing positive for SARS-CoV-2

The number of deaths were low in both the CVID and the control groups (≤2 and 4, respectively), with no difference in mortality rates between the two groups (**Table 2**).

### Vaccine effects

Compared to individuals who had received two SARS-CoV-2 vaccinations, those vaccinated three times had slightly lower risk of SARS-CoV-2 infection; however, the difference was not significant [IRR 0.8 (95% CI, 0.5–1.3)] (**Table 3**). The risk of hospital contact was increased for CVID individuals, who had received three *vs*. two SARS-CoV-2 vaccination; due to the low numbers, the IRR estimate is, however, uncertain with wide confidence intervals [IRR 2.7 (95% CI, 0.7–11.4)] (**Table 3**).

**Table 3:** Vaccine effectiveness.

	CVID	Time to the event	Unadjusted IRR 95% Cl)	Adjusted IRR* (95% Cl)
Time to f irst positive test following vaccination status time-updated		PYR		
Two vaccines1	23	29.8	Ref (1)	Ref (1)
Three vaccines 1	119	82.6	0.8 (0.5-1.3)	1.0 (0.6-1.6)
Time to f irst hospital contact following vaccination status time-updated				
Two vaccines1	2	38.0	Ref (1)	Ref (1)
Three vaccines 1	36	109.0	2.7 (0.7-11.4)	2.3 (0.5-10.0)

CVID, Common variable immunodeficiency; PYR, Person-years of follow-up; IRR, Incendent Rate Ratio, 95% CI: Confidence Interval.

^1^ Calendar time as time axis.

*Adjusted for age, gender and Charlson’s Comorbidity Index(Low=0, Medium=1-2, High =>2).

## Discussion

In this Danish nationwide matched cohort study, we compared COVID-19 outcomes among individuals with CVID to that of an age- and gender-matched cohort from the general population. Despite early vaccination and high coverage, we found a slightly higher risk of both first and second SARS-CoV-2 infection among individuals with CVID; however, this was mainly seen after 1 January 2022. The risk of hospital contact and hospitalizations was likewise increased in the CVID group but, reassuringly, the need of mechanical ventilation and mortality was not higher in the CVID cohort. The overall benign course of infection found was in spite of higher Charlson comorbidity scores among the CVID patient. The difference in Charlson comorbidity scores between the individuals with CVID and the control group might contribute to the mildly worse outcome among the CVID patients. Furthermore, part of the increased comorbidity score among the CVID patients may be induced by their immune deficiency. The relative benign course of SARS-CoV-2 infection among the individuals with CVID was also in spite of the weaker immunological vaccination responses reported among individuals with CVID (8, 31).

The SARS-CoV-2 vaccination program in Denmark (as elsewhere) was initially prioritized to individuals anticipated to be at increased risk of a serious course of COVID-19, with a subsequent expansion to individuals at anticipated lower risk. As age was very early in the epidemic identified as a significant risk factor for severe COVID-19 (32), it is not surprising that the CVID group, with a mean age of 48 years, received vaccination earlier than the age-matched control group.

It could be speculated that the higher risk of SARS-CoV-2 infection in the CVID group might be due to higher testing frequency. Very early in the epidemic, SARS-CoV-2 testing was restricted to individuals with relevant exposure and those with marked symptom, but soon, testing became widely accessible and it is therefore unlikely that a differential testing strategy would fully explain the differences. However, analysis of testing frequency in the two groups did reveal an increased testing frequency among individuals with CVID early in the epidemic (**Appendix Figure 1A**). However, during the spring of 2021, the pattern reversed, most likely representing the requirements for a negative SARS-CoV-2 test for many work- and social event-related activities. During this period, the CVID group might have abstained from these activities (i.e., worked from home and/or reduced participation in social events) to a higher degree relative to the individuals from the population control. During the latter part of the study period, there was no discernible difference in testing pattern, probably reflecting the reversion to a pre-SARS-CoV-2-like lifestyle.

In addition to the CVID group being included in the accelerated vaccination program, individuals at perceived risk of severe infection were recommended to protect themselves as much as possible. Despite these precautions, the IRR for both first and subsequent SARS-CoV-2 infections was, as mentioned, 1.3 and 2.1, respectively, pointing to the high transmissibility of SARS-CoV-2. The increased risk was mainly observed after 1 January 2022, when omicron was the dominant strain (33), many restrictions were removed, and a potential change in behavior was observed probably due to the perceived change in risk associated with infection with omicron.

The finding of increased risk for testing positive for SARS-CoV-2 in the CVID group concurs with those reported in a Danish study investigating the risk of SARS-CoV-2 infections among solid organ transplant recipients relative to a matched control group from the general population (24). Other studies have also reported the protective strategies including shielding to be less effective than hoped for (34).

Though the CVID group had an overall higher relative risk SARS-CoV-2 associated hospital contact and admission with severe COVID-19, the absolute risk of admission with severe COVID-19 was low and risks of mechanical ventilation and death were not substantially increased relative to the general population. For both outcomes, the numbers were very small, and the estimates are therefore uncertain. CVID patients suffer from increased risk of upper respiratory symptoms, which may be misclassified as COVID-19. We therefore evaluated risk of hospitalization irrespective of diagnosis and found a more than fourfold increased risk of hospitalization in the CVID population after a positive SARS-CoV-2 test, which ensures that the increased risk of hospitalization does not stem from misclassification of diagnoses.

The strategy with earlier vaccination to the CVID group might have contributed to the relative benign course of infection. It has previously been shown that SARS-CoV-2 vaccination offers good protection against severe COVID-19 (35, 36). We presume that the threshold for hospital contact and admission for individuals with CVID, with a positive SARS-CoV-2 test, could be lower relative to that of the general population. Furthermore, during the latter part of the study period, individuals with CVID could, as per national Danish guidelines (37), receive monoclonal antibodies directed against SARS-CoV-2. The usage of monoclonal antibodies for this indication was, however, limited. Furthermore, in Denmark, the monoclonal antibody used during this period was Sotrovimab^®^, which has been shown to have reduced activity against the omicron subvariants (38, 39). It is therefore unlikely to have contributed considerably to our outcome findings but probably contributes to the finding of large differences in risk of hospital contact and hospitalization.

We were unable to show better protection from three versus two vaccinations. This might be due to the few serious infections in both study populations. The added value of the third vaccination is mainly the increased protection against severe COVID-19 (36). Furthermore, the change in viral strains might have contributed to this finding; the added protection from a third vaccine is reduced, when the omicron strain prevails (40). In Denmark the omicron rapidly replaced the delta variant from 21 November and over the ensuing months (33). However, also with predominance of the omicron lineage, a third vaccination has been shown to increase the protection from severe COVID-19 (40). However, we cannot exclude a degree of confounding in the sense that a third vaccine was offered earlier to those most at risk for severe SARS-CoV-2 infection.

The overall benign outcome in the CVID group is reassuring for the patients and in line with our previous study (20) and with several other reports (5, 21). However, other studies have reported more severe courses of infection among CVID patients. An Italian study reported a 22% hospitalization rate among CVID patients (16). The study included individuals with various IEI and both children and adults, with patients with CVID being the largest patient group (16). Though the overall mortality rate was similar to that of the background population, the age at death was significantly lower (all deaths were among adult patients) (16). In the study by Shields et al. (8), overall high rates of both hospitalizations and deaths were reported for patients with both primary (PID) and secondary immunodeficiencies (SID), with the latter faring worse than the PID patients. The included CVID group also had high hospitalization and mortality (35%) rates (8). The UK study, however, included only 23 CVID patients and was conducted very early in the epidemic (8), possibly explaining the much worse outcome in that study population relative to the current study. Early in the epidemic, shortage of testing equipment and limitation in accessibility as well as reluctance to visit health facilities might have led to underdiagnosis among individuals with mild SARS-CoV-2 infection.

CVID is likely a group of related conditions rather than one distinct disease entity (41). Likewise, the complications among patients with CVID differ, which could affect the risk of severe SARS-CoV-2 infection. Milito et al. (42) found a higher risk for severe SARS-CoV-2 infection among individuals with CVID-related lung complications relative to CVID patients without.

Relative to the other studies, the strengths of the current study are as follows: the focus on a single IEI; our ability to conduct a nationwide, population-based study with the inclusion of an age- and gender-matched control group; and our relatively large study group. Furthermore, our access to national data allowed us to follow the two cohorts for the complete period of the Danish SARS-CoV-2 epidemic. To the best of our knowledge, this is the largest study to investigate the course of SARS-CoV-2 infection among a specified IEI.

Our study also has some limitations. We identified the CVID patients from the national hospital databases as individuals with ≥3 ICD-10 codes, which are in Denmark generally used to categorize individuals with CVID. We have previously shown that including more ICD-10 codes from the broader headings of “certain disorders involving the immune mechanism (D80–D89)” is unlikely to identify patients with CVID (43). We chose to limit the population to those with the relevant diagnoses registered on several occasions as a substantial fraction of the individuals only registered with the diagnoses once or twice probably represent misregistration. These codes might be used at visits for suspected immunodeficiency, with the diagnosis subsequently being modified.

The size of the CVID population identified by this method is in line with the estimated CVID population in Denmark (44).

In summary, using Danish national data, we found an overall benign course of SARS-CoV-2 infection among individuals with CVID.

## Data availability statement

The raw data supporting the conclusions of this article will be made available by the authors, without undue reservation.

## Ethics statement

The study was approved by The Danish Data Protection Agency (permission no 21/04383). According to Danish regulations, national surveillance activities as well as studies solely relying on register information, do not require individual consent nor approval from an ethics committee. Written informed consent for participation was not required for this study in accordance with the national legislation and the institutional requirements.

## Author contributions

TLK, LDR, CSL, A-BH, MS and LSK, constituting the Danish Primary Immunodeficiency guidelines group, conceived the idea for the study in close collaboration with NO. The study plan was finalized by TLK, LDR, and NO. NO accessed and verified the underlying study data. NO performed the statistical analyses. TLK wrote the first manuscript draft. All the authors had full access to the data and contributed to interpreting the data and writing the manuscript. All authors contributed to the article and approved the submitted version.

## Conflict of interest

The authors declare that the research was conducted in the absence of any commercial or financial relationships that could be construed as a potential conflict of interest.

## Publisher’s note

All claims expressed in this article are solely those of the authors and do not necessarily represent those of their affiliated organizations, or those of the publisher, the editors and the reviewers. Any product that may be evaluated in this article, or claim that may be made by its manufacturer, is not guaranteed or endorsed by the publisher.
