# Supplementary figures and images for: Outcome of SARS-CoV-2 infection among patients with common variable immunodeficiency and a matched control group: A Danish nationwide cohort study
Source: Front Immunol. 2022 Sep 23;13:994253. doi: 10.3389/fimmu.2022.994253 (PMC9539828; doi:10.3389/fimmu.2022.994253)

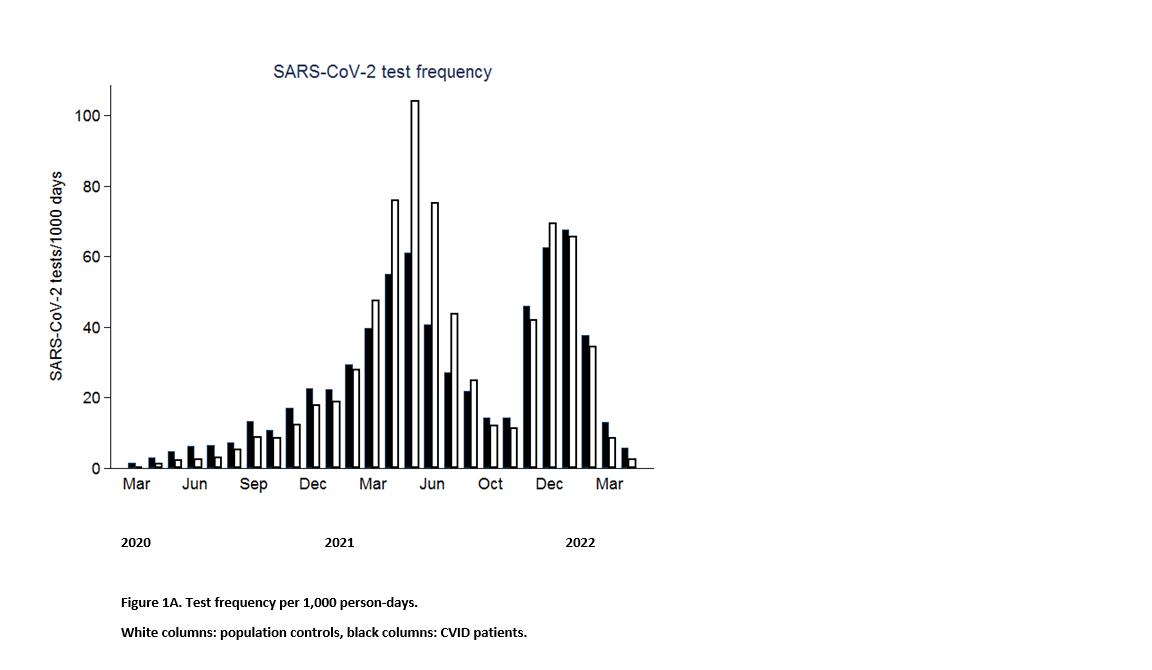

Supplement: Supplementary file 1 [file Image_1.tif]
